# Neural basis for pheromone signal transduction in mice

**DOI:** 10.3389/fncir.2024.1409994

**Published:** 2024-04-29

**Authors:** Ken Murata, Takumi Itakura, Kazushige Touhara

**Affiliations:** ^1^Laboratory of Biological Chemistry, Graduate School of Agricultural and Life Sciences, Department of Applied Biological Chemistry, The University of Tokyo, Tokyo, Japan; ^2^Division of Biology and Biological Engineering, TianQiao and Chrissy Chen Institute for Neuroscience, California Institute of Technology, Pasadena, CA, United States

**Keywords:** pheromone, vomeronasal system, hypothalamus, innate behavior, neural circuit

## Abstract

Pheromones are specialized chemical messengers used for inter-individual communication within the same species, playing crucial roles in modulating behaviors and physiological states. The detection mechanisms of these signals at the peripheral organ and their transduction to the brain have been unclear. However, recent identification of pheromone molecules, their corresponding receptors, and advancements in neuroscientific technology have started to elucidate these processes. In mammals, the detection and interpretation of pheromone signals are primarily attributed to the vomeronasal system, which is a specialized olfactory apparatus predominantly dedicated to decoding socio-chemical cues. In this mini-review, we aim to delineate the vomeronasal signal transduction pathway initiated by specific vomeronasal receptor-ligand interactions in mice. First, we catalog the previously identified pheromone ligands and their corresponding receptor pairs, providing a foundational understanding of the specificity inherent in pheromonal communication. Subsequently, we examine the neural circuits involved in processing each pheromone signal. We focus on the anatomical pathways, the sexually dimorphic and physiological state-dependent aspects of signal transduction, and the neural coding strategies underlying behavioral responses to pheromonal cues. These insights provide further critical questions regarding the development of innate circuit formation and plasticity within these circuits.

## Introduction

Animals utilize chemosensory signals, which are crucial for mediating a range of behaviors involved in survival and reproduction (Wyatt, 2014). These signals are encapsulated in biochemical compounds known as pheromones, which facilitate intraspecific communication (Karlson and Lüscher, 1959). Pheromones are principally detected by two sensory systems: the main olfactory system and the vomeronasal system (Baum and Cherry, 2015; Tirindelli, 2021). The main olfactory system is responsible for detecting volatile pheromones, while the vomeronasal system is particularly attuned to decoding social cues embedded within these signals (Holy, 2018). Such cues encompass a range of biological information, including species, sex, developmental stage, health status, and reproductive condition of conspecifics. A variety of vomeronasal ligands carrying this intricate information have been identified, indicating a complex and diverse chemosensory communication network among animals (Murata and Touhara, 2021). Vomeronasal receptors (VRs), which are a subset of the G protein-coupled receptor (GPCR) superfamily, are specialized for the detection of these chemosensory signals (Silva and Antunes, 2017). These receptors translate the chemical information from ligands into a biological response through dedicated signal transduction pathways, ultimately leading to behavioral or physiological responses.

### Vomeronasal ligands, receptors, and functions

**Figure 1:**
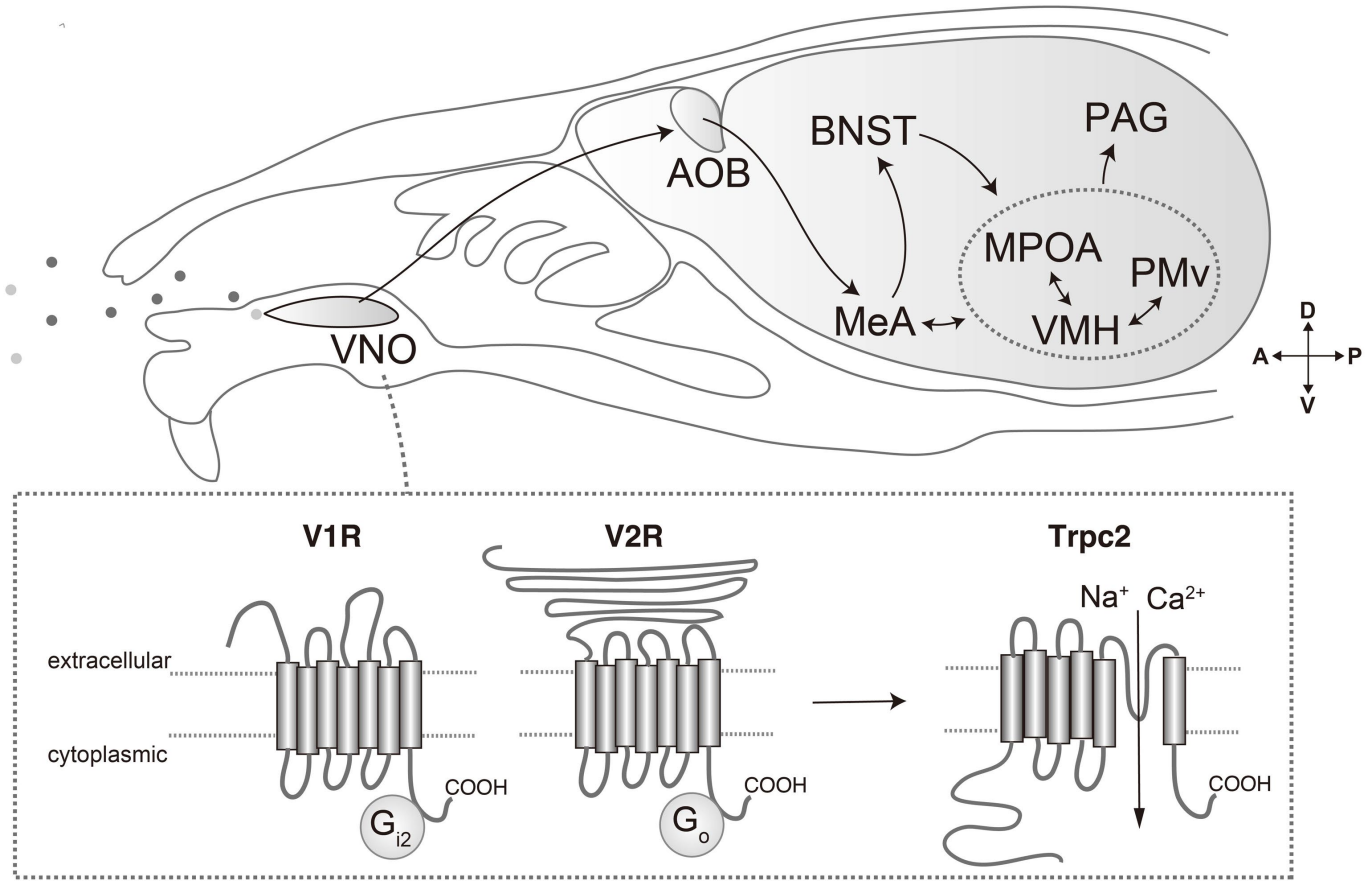
Pheromone molecules are received by vomeronasal receptors expressed in vomeronasal organ (VNO). Vomeronasal receptors are mainly categorized into V1R and V2R, which are coupled with Gi2 and Go, respectively. Activation of receptors results in opening of transient receptor potential channel 2 (Trpc2), generating action potentials. The signals are transmitted to the accessory olfactory bulb, and then to the medial amygdala (MeA) and bed nucleus of the stria terminalis (BNST). From there, pheromone signals are transmitted to the hypothalamus, such as medial preoptic area (MPOA), ventromedial hypothalamus (VMH) and ventral premammillary nucleus (PMv). From the hypothalamus, processed information is conveyed to downstream motor effector areas through the mesencephalic periaqueductal gray (PAG).

VRs are expressed in vomeronasal sensory neurons (VSNs) of the vomeronasal organ (VNO): a tubular organ located in the base of the nasal septum (Døving and Trotier, 1998). These receptors are categorized into two principal classes: V1Rs and V2Rs (Figure 1). Mice have approximately 240 V1R genes and 120 V2R genes (Young and Trask, 2007; Miller et al., 2020). Each of these receptor types has a distinct structure and function. V1Rs are members of the rhodopsin-type GPCR family and are typically coupled with the G protein Gαi2 (Trouillet et al., 2019). They are generally involved in detecting small molecule ligands, such as volatile compounds and steroid derivatives (Lee et al., 2019; Wong et al., 2020). In mice, V1Rs are expressed in the apical layer of the VNO and are responsible for recognizing a variety of urinary molecules that convey information about sex and physiological states (Doyle and Meeks, 2018). V2Rs belong to the class C GPCR family and are associated with the G protein Gαo (Chamero et al., 2011). V2Rs have a large extracellular domain that is thought to be involved in ligand recognition. They are primarily expressed in the basal layer of the VNO and are tuned to detect larger molecules such as peptides and proteins (Pérez-Gómez et al., 2014).

Each VSN expresses only one or a restricted few VRs, which allows for a highly specific response to particular chemosensory cues (Nikaido, 2019). When a VR binds to its ligand, it initiates a cascade of intracellular events that lead to the opening of ion channels, and ultimately results in the generation of action potentials (Yu, 2015). These electrical signals are then transmitted to the brain, where they are processed into behavioral responses.

The transient receptor potential channel 2 (Trpc2) is specifically expressed in the VNO and plays a critical role in signal transduction (Figure 1). The disruption of Trpc2 significantly diminishes the responsiveness of VSNs, leading to marked alterations in a spectrum of social behaviors, such as inter-male and maternal aggression, basically because of impairment of sex recognition (Leypold et al., 2002; Stowers et al., 2002; Kimchi et al., 2007). The role of Trpc2 in parental behavior is somewhat controversial. In one report, Trpc2-KO virgin male mice show marked reduction in pup-directed aggression, and even exhibit parental care (Wu et al., 2014). Conversely, other research underscores the relevance of Trpc2 in maternal behaviors (Fraser and Shah, 2014). Moreover, it is currently unclear whether the phenotypic manifestations observed in Trpc2-KO models are solely attributable to compromised vomeronasal signaling or whether they may also be a consequence of perturbations in the developmental processes. Indeed, some studies have implied the significance of the vomeronasal input during the development in terms of gene expression, anatomy, and behaviors (Cross et al., 2021; Pfau et al., 2023).

The ligands for vomeronasal receptors are diverse and are often species-specific molecules involved in social communication. A prominent class of these ligands is the exocrine gland-secreting peptide (ESP) family (Kimoto et al., 2005). This group comprises species-specific peptide ligands that have undergone evolutionary divergence from the α-globin gene in rodent species (Niimura et al., 2020). ESPs are synthesized and secreted by various exocrine glands and are subsequently detected by a subset of the V2R receptor class (Kimoto et al., 2007). The differential secretion patterns of ESPs by sex and genetic strain enable the transmission of critical information regarding an individual’s sex and unique identity. One notable peptide, ESP1, secreted by the extraorbital lacrimal gland of male mice, is received by Vmn2r116 and enhances female sexual receptivity and inter-male aggression (Haga et al., 2010; Hattori et al., 2016). Conversely, ESP22, found in the tear fluid of juvenile mice, acts through Vmn2r115—a receptor closely homologous to Vmn2r116— and suppresses sexual behavior in both adult male and female mice (Ferrero et al., 2013; Osakada et al., 2018).

Another significant class of vomeronasal ligands is Major Urinary Proteins (MUPs), primarily found in the urine of rodents (Hurst and Beynon, 2004). MUPs bind to and gradually release volatile molecules that are detected by the V1Rs (Leinders-Zufall et al., 2000). Concurrently, MUPs themselves act as agonists for V2Rs (Chamero et al., 2007). Since each mouse strain secretes specific patterns of MUPs and males release more MUPs than females, MUPs are implicated in behaviors associated with territorial demarcation and individual recognition (Roberts et al., 2012). Notably, MUP3 and MUP20 have been identified as having an aggression-promoting effect in mice (Kaur et al., 2014). Although the V2Rs for MUPs have yet to be identified, electrophysiological recordings from putative V2R-expressing VSNs have suggested that a diverse array of V2R subtypes interact with MUPs in a complex manner: some demonstrating specificity to individual MUPs, while others respond to multiple MUP isoforms. The mechanism by which this combinatorial receptor coding is interpreted by the downstream neural circuits is not understood.

Urine is a major source of vomeronasal ligands. The urine of female mice is rich in sulfated steroids, which are hormonal derivatives that act as ligands for V1Rs (Nodari et al., 2008; Isogai et al., 2011). Some VRs have been identified to bind sulfated steroids in a combinatorial manner. The presence of these V1R ligands in female urine may convey information regarding the female’s reproductive status, such as estrous cycle phase, thereby enhancing sexual behavior in the male mouse (Haga-Yamanaka et al., 2014). On the other hand, the identification of VRs responsive to male urine has not been as fruitful. Neurons expressing Vmn2r53 in the VNO are activated by urine from males across different mouse strains (Itakura et al., 2022). Activation of Vmn2r53 has been associated with the promotion of inter-male aggressive behavior. The consistent activation of Vmn2r53 by urine from various strains of male mice suggests that its ligand is a robust marker of ‘maleness’ within the species.

Ligands for the vomeronasal system are not limited to species-specific molecules. For instance, hemoglobin from diverse species acts as a ligand for Vmn2r88 and has been shown to elevate digging and rearing behaviors in lactating female mice, although the ethological significance of these behaviors remains undetermined (Osakada et al., 2022).

The submandibular gland protein C (Smgc) from pups and female mice has been identified as a ligand for Vmn2r65 (Isogai et al., 2018). In virgin male mice, Vmn2r65, alongside Vmn2r88, appears to be partially required for the exhibition of infanticidal behavior. It is postulated that activation of certain vomeronasal receptor pairs is essential for the induction of infanticidal behavior, yet the precise receptor combinations that are sufficient for eliciting such behavior have not been fully elucidated.

The neural encoding of signals from VSNs expressing narrowly-tuned VRs is hypothesized to be interpreted by downstream neural circuits in a manner akin to a labeled-line model (Tye, 2018). The elucidation of this mechanism is anticipated to significantly enhance our understanding of the neural bases of innate behaviors. Further analysis of these neural circuits may also yield valuable insights into the general principles governing sensory information processing and the resultant behavioral manifestations.

### Neural basis for vomeronasal signal transduction

The vomeronasal system is characterized by its distinct neural pathway through which sensory information from the VNO is transmitted to the hypothalamus (Figure 1) (Halpern, and Martínez-Marcos, 2003; Ishii and Touhara, 2019). The initial synaptic relay occurs at the accessory olfactory bulb (AOB), where the axons of VSNs responding to pheromonal stimuli converge upon multiple glomeruli (Mombaerts, 2004; Dulac and Wagner, 2006). Here, they establish synaptic connections with second-order neurons: mitral and tufted cells. These AOB neurons integrate VR signals to encode sexual and species-specific information (Hammen et al., 2014). Subsequently, this integrated signal is propagated to higher brain areas such as the medial amygdala (MeA) and the bed nucleus of the stria terminalis (BNST), which are pivotal in modulating socio-sexual behaviors through the processing of species- and sex-specific cues (Li et al., 2017; Yang et al., 2022).

The information is further transmitted from the MeA and BNST to the interconnected hypothalamic nuclei implicated in reproductive and aggressive behaviors: the ventromedial hypothalamus (VMH), medial preoptic area (MPOA), and ventral premammillary nucleus (PMv) (Choi et al., 2005). These hypothalamic neurons are characterized by distinctive patterns of gene expression and neural circuitry, which confer selective responsiveness to various stimuli. The functional specialization of these neuronal populations has been elucidated through various experimental methodologies, such as *in vivo* neural activity recording and the application of optogenetic or chemogenetic techniques, which have substantiated the unique role of these neurons in behavioral modulation. Notably, by leveraging Ca^2+^ imaging, neurons responsive to male and female signals are shown to be largely segregated from the VNO to the VMH (He et al., 2008; Hammen et al., 2014; Li et al., 2017; Remedios et al., 2017; Karigo et al., 2021; Yang et al., 2022).

The activity of the hypothalamic nuclei extends to the midbrain periaqueductal gray (PAG), a key structure in orchestrating various survival behaviors. The PAG integrates these signals and extends projections to additional brain regions responsible for the execution of motor functions (Falkner et al., 2020; Chen et al., 2021). This forms a comprehensive neural circuit that translates the detection of pheromonal signals into appropriate behavioral responses.

Recent studies have provided significant insights into the neurobiological underpinnings of intermale aggression. Specifically, a cluster of neurons located within the ventrolateral part of the VMH (VMHvl), which express estrogen receptor type 1 (Esr1) and are henceforth referred to as VMHvl^Esr1^ neurons, have been identified as critical mediators of intermale aggressive behavior (Lin et al., 2011; Lee et al., 2014). The activity and sensitivity of VMHvl^Esr1^ neurons are dynamically modulated by sexual experience, which in turn affects the neural coding of sexual cues and can lead to the onset of aggressive responses (Remedios et al., 2017).

In female sexual behavior, a complex interaction between hormonal fluctuations and pheromonal cues is important. A subset of VMHvl^Esr1^ neurons, which express Cckar but not NPY2r, are both necessary for the initiation of and sufficient to induce sexual receptivity in females (Knoedler et al., 2022; Liu et al., 2022; Yin et al., 2022). This subset of neurons exists only in females and exhibits an increase in axonal projections to the anteroventral periventricular nucleus and changes neurophysiological properties during the estrous cycle (Inoue et al., 2019; Knoedler et al., 2022). These findings highlight the essential role of hormone-sensitive neurons within the VMHvl in regulating female sexual behaviors.

**Figure 2:**
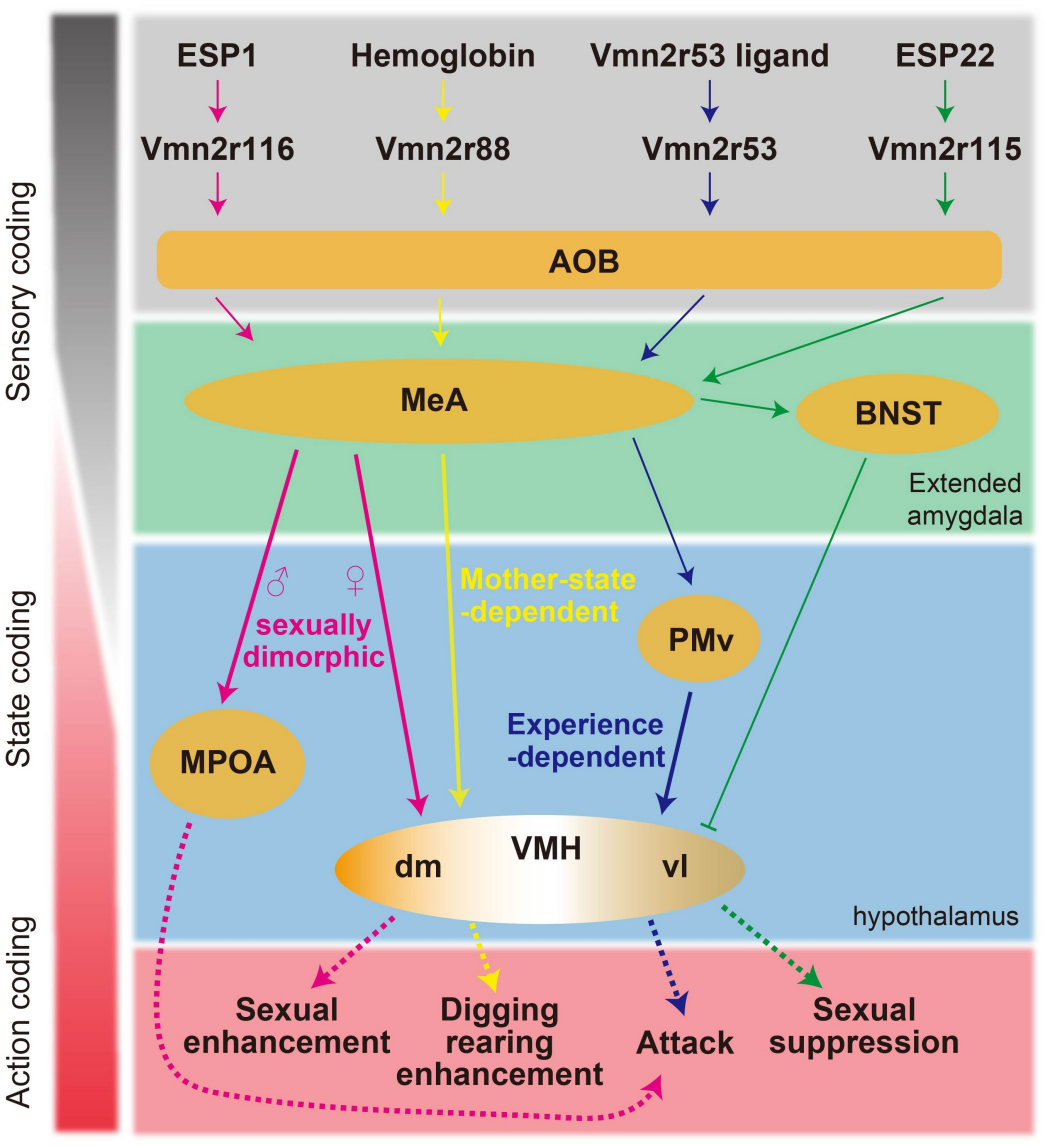
Schematic summary of the proposed neural circuits responsible for pheromone-mediated behaviors in mice.

ESP1 has been shown to enhance female sexual receptivity, with its neural circuitry being extensively characterized (Figure 2) (Ishii et al., 2017). ESP1 is detected by Vmn2r116-positive VSNs, leading to the activation of the caudal part of the AOB. This triggers a cascade of neural activity that results in the activation of glutamatergic neurons within the MeA. Notably, the propagation of this neural signal does not proceed to the VMHvl as might be expected, but rather to the VMH dorsomedial part (VMHdm)—a region typically implicated in fear response modulation. Within the VMHdm, a subpopulation of neurons expressing the nuclear receptor steroidogenic factor 1 (VMHdm^SF1^) is preferentially responsive to ESP1. These neurons exhibit a response profile that is distinct from that of neurons responding to predator odors within the same neural cluster. Experimental optogenetic reactivation of the ESP1-responsive VMHdm^SF1^ neurons can induce an increase in sexual receptivity akin to that observed naturally in response to ESP1. Conversely, the genetic disruption of VMHdm^SF1^ neurons leads to a diminution of the ESP1-induced enhancement in sexual receptivity, although basal levels of receptivity are unaffected. In stark contrast, the ablation of VMHvl^Esr1^ results in the complete abolition of sexual receptivity.

Conversely, ESP22 suppresses female sexual behaviors. This suppressive influence of ESP22 is mediated through GABAergic projections from the BNST to the VMHvl (Osakada et al., 2018).

The signaling mechanisms of ESP1 and ESP22 exemplify a labeled-line neural circuit model; however, pheromonal neural circuitry does not universally adhere to such a linear pathway (Li et al., 2017; Li and Dulac, 2018).

Neural mechanisms of pheromone-mediated intermale aggression have also been characterized by focusing on specific pheromones and receptors. Exposure to a pheromonal component that activates Vmn2r53 invariably results in the activation of progesterone receptor (PR)-expressing neurons in the PMv (PMv^PR^) in male mice (Itakura et al., 2022). This response occurs regardless of previous social interactions. It is noteworthy that although male urine is composed of a multitude of pheromonal substances in addition to Vmn2r53 ligands, the bulk calcium responses—serving as proxies for neural activity—elicited by this individual pheromonal component are quantitatively analogous to those triggered by the complete male urine. Contrastingly, after experiencing aggression, this pheromone fraction activates PR-expressing neurons in the VMHvl (VMHvl^PR^), which substantially overlap with the VMHvl^Esr1^ neurons. In addition, the magnitude of this response is much smaller compared to the response elicited by exposure to complete male urine. Chemogenetic suppression of PMv^PR^ function abolishes VMHvl^PR^ responses to this specific pheromonal fraction and attenuates responses to male urine. This finding implicates PMv^PR^ neurons as a pivotal upstream mediator to the VMHvl^PR^ in the neural processing of male pheromone signals. Remarkably, Vmn2r53 knockout significantly diminishes PMv^PR^ responses to male urine, suggesting an overrepresentation of Vmn2r53-mediated signaling within the PMv^PR^. In contrast, strain-specific male pheromones such as ESP1 and MUP3, which are also implicated in the induction of aggressive behavior, do not appear to activate PMv^PR^ or VMHvl^PR^ neurons. In males, ESP1 stimulates excitatory neurons in the MeA and downstream neurons in the BNST and MPOA (Ishii et al., 2017). However, the direct causal link between the activation of these neural pathways and the potentiation of aggressive behavior remains to be conclusively determined. These studies imply the existence of redundant or parallel pathways for pheromone-mediated aggression.

State-dependent signal transduction of hemoglobin is observed. Hemoglobin activates Vmn2r88, mediated by its interaction site, Gly17, on the hemoglobin (Osakada et al., 2022). The hemoglobin signal reaches the MeA in mice, regardless of sex. However, VMHdm is selectively activated in lactating females. As a result, in lactating mothers, hemoglobin enhances digging and rearing behaviors. Manipulation of VMHdm^SF1^ neurons is sufficient to induce the hemoglobin-mediated behaviors.

Collectively, the identification of specific pheromone molecules and corresponding receptors responsible for behavioral regulations has contributed to delineating neural circuits from sensory input to behavioral output since this approach simplifies input–output relations (Figure 2).

### Perspective

Research on pheromones and their behavioral impacts provides a rich field, particularly with regard to the formation and function of neural circuits inducing innate behaviors, known as ‘labeled line’ circuits. A viable starting point for dissecting these pathways is the study of VRs with identified pheromone ligands. For example, the closely related receptors Vmn2r115 and Vmn2r116 bind to ESP22 and ESP1, respectively, yet exert opposite effects on female sexual behavior (Haga et al., 2010; Osakada et al., 2018). What variations exist in the spatial distribution of axonal terminals within the AOB’s glomerular array among VSNs expressing different VRs? Do VRs dictate projection patterns to the AOB in a manner similar to olfactory receptors? How do VSNs expressing individual VRs form synapses with mitral/tufted cells? This includes identifying whether there are distinct subtypes within the mitral/tufted cell populations based on their connectivity and gene expression. Furthermore, do mitral/tufted cells form specific synaptic connections with neurons in the MeA that correspond to discrete valences? If so, how is this multisynaptic ‘labeled line’ wired? Elucidating these mechanisms is crucial for a comprehensive understanding of the development of innate neural circuits.

The investigation into the mechanisms of sexually dimorphic circuit formation is a significant area of research within neurobiology (Yang and Shah, 2014). One promising avenue is the study of pheromone signal transduction pathways. Notably, sexually dimorphic processing of information is observed in response to ESP1, which elicits sexually divergent activations in the MeA. A critical question arises: at what developmental stages and by what processes does this sexual dimorphism manifest? Current evidence suggests that the sexually dimorphic responses observed in the MeA are heavily influenced by sex hormone signaling. This is supported by findings that male mice deficient in the enzyme aromatase, which is crucial for estrogen synthesis, exhibit disrupted sexually dimorphic responses (Bergan et al., 2014). It is hypothesized that the pathway from the MeA to the MPA or VMHdm in response to ESP1 may operate through a regulatory mechanism similar to that governed by sex hormone signaling. This pathway represents an excellent model for elucidating the precise mechanisms underlying sexually dimorphic neural circuit formation and pheromonal information processing.

How the pheromone molecule, its receptor, and subsequent signaling pathways co-evolved is an interesting question. The ESP and MUP gene families are pivotal examples, having undergone diversification predominantly via gene duplication—a well-documented mechanism in evolutionary biology (Sheehan et al., 2019; Niimura et al., 2020). Similarly, vomeronasal receptors have also evolved through this mechanism. Despite similarities within the ESP and MUP families and their receptors, there are significant functional divergences among their members, making them excellent models for studying pheromone evolution. In both insect pheromone systems and mammalian taste systems, which are well-known examples of labeled line signal transduction, the type of cell expressing the receptor determines the perceived value of the ligand (Mueller et al., 2005; Zhao and McBride, 2020). However, it remains uncertain whether a similar paradigm applies to the mammalian vomeronasal system, or whether ligand valuation is an intrinsic property of the receptor. Indeed, in the mammalian olfactory system, swapping one receptor gene for another affects the projection pattern of the given sensory neurons, which would affect the perception of the odorant, suggesting that the receptor gene itself has a deterministic factor of the value of the corresponding ligand (Mombaerts et al., 1996). This query could be empirically addressed for the vomeronasal system through targeted genetic experiments involving the Vmn2r115 and Vmn2r116 receptors. For instance, an experimental strategy might include the disruption of the Vmn2r116 gene and the substitution of its sequence at the Vmn2r115 locus to assess the functional outcomes on ESP1-mediated behaviors—specifically, whether it continues to facilitate sexual behavior or shifts to inhibiting such behavior, akin to the effect observed with ESP22. Should the former occur, it would suggest a decisive role of receptor functionality; conversely, the latter outcome would indicate a locational influence on gene function. The identification and comprehensive characterization of receptors for ESPs and MUPs remain incomplete. Further elucidation of the evolutionary trajectories of these ligands and their corresponding receptors could significantly enhance our understanding of molecular evolution of pheromone-mediated communication. Additionally, investigating the mechanisms through which signals from these receptors interact and integrate with those from other sensory modalities to elicit specific behavioral responses presents an intriguing area of research.

The signal transduction pathways mediated by ESP1 and ESP22 appear to support a labeled line coding system; however, tracking the neural response toward conspecific sex across different social experiences revealed that pheromone signal processing involves complex mechanisms beyond this simple paradigm. Recent studies have indicated that sexual experiences can alter the neural coding of conspecific sex cues within the MeA and VMHvl^Esr1^. The use of various VR and ligand pairs may be instrumental in elucidating the precise mechanisms involved, given that the inputs derived from these receptor-ligand interactions are typically simple and stable. For instance, the signal transduction pathway involving Vmn2r53, which is influenced by aggressive behavioral experiences, might serve as an excellent model for studying synaptic plasticity between the VMHvl and the PMv.

A variety of aggression-inducing pheromones have been identified; however, the underlying mechanisms through which these pheromones collectively influence aggressive behaviors are not yet fully understood. Notably, the MUPs such as MUP3 and MUP20, as well as ESP1, exhibit secretion patterns that are distinct among mouse strains. In contrast, Vmn2r53 is responsive to a male-specific pheromone present in the urine across various male mouse strains, indicating that Vmn2r53 may detect a ubiquitous male signal within the species. These findings could be pivotal for the discussion on how differentially processed pheromone signals can orchestrate uniform behavioral responses. Moreover, the interaction complexity of pheromone signals is further exemplified by certain behaviors, such as infanticide, which are elicited only in the presence of multiple pheromonal signals. Identifying the precise pheromonal blend required to initiate such complex behaviors remains an outstanding challenge in the field. Furthermore, elucidating the specific sites and mechanisms through which the vomeronasal system processes and integrates these pheromone signals is an essential area of research that warrants further investigation.

The perception of pheromones alone typically does not elicit specific behavioral responses. The presence of conspecifics and the assimilation of diverse sensory inputs, including auditory, visual, and tactile information, is requisite. Elucidating how pheromone signaling is integrated with other sensory modalities is essential for a thorough understanding of behavioral expression. This intersection of sensory integration and behavior is a pivotal area for future investigation.

The framework of sensory input to aggressive and sexual behavioral output is emerging owing to the characterization of pheromone molecules and corresponding receptors along with the detailed functional analysis within the central nervous systems. In contrast, we scarcely know how pheromones trigger physiological responses even though these phenomena are classically reported in rodents. Since the 1950s, several pheromone-mediated phenomena that modulate reproductive function in mice have been reported. Estrus cycle is suppressed in group housed females (van der Lee and Boot, 1955), and male urine induces estrus in those females (Whitten, 1956). Male urine also accelerates puberty in female mice (Vandenbergh, 1967). Exposure to a novel male induces pregnancy failure in female mice (Bruce, 1959). The advent of modern neuroscientific methodologies, including cell-type-specific imaging and sensors for neuromodulators, offers promising avenues for deciphering the central mechanisms governing these phenomena. In summary, ongoing research into pheromonal communication is poised to yield profound insights into the neural circuitry that orchestrates behavioral and physiological adaptations.

## Author contributions

KM: Conceptualization, Data curation, Investigation, Writing – original draft, Writing – review & editing. TI: Conceptualization, Data curation, Writing – original draft, Writing – review & editing. KT: Conceptualization, Funding acquisition, Project administration, Writing – original draft, Writing – review & editing.

## References

[ref1] Baum M. J. Cherry J. A. (2015). Processing by the main olfactory system of chemosignals that facilitate mammalian reproduction. Horm. Behav. 68, 53–64. doi: 10.1016/j.yhbeh.2014.06.003, PMID: 24929017

[ref2] Bergan J. F. Ben-Shaul Y. Dulac C. (2014). Sex-specific processing of social cues in the medial amygdala. eLife 3:e02743. doi: 10.7554/eLife.02743, PMID: 24894465 PMC4038839

[ref3] Bruce H. M. (1959). An exteroceptive block to pregnancy in the mouse. Nature 184:105. doi: 10.1038/184105a0, PMID: 13805128

[ref4] Chamero P. Katsoulidou V. Hendrix P. Bufe B. Roberts R. Matsunami H.. (2011). G protein G(alpha)o is essential for vomeronasal function and aggressive behavior in mice. Proc. Natl. Acad. Sci. USA 108, 12898–12903. doi: 10.1073/pnas.1107770108, PMID: 21768373 PMC3150917

[ref5] Chamero P. Marton T. F. Logan D. W. Flanagan K. Cruz J. R. Saghatelian A.. (2007). Identification of protein pheromones that promote aggressive behaviour. Nature 450, 899–902. doi: 10.1038/nature05997, PMID: 18064011

[ref6] Chen J. Markowitz J. E. Lilascharoen V. Taylor S. Sheurpukdi P. Keller J. A.. (2021). Flexible scaling and persistence of social vocal communication. Nature 593, 108–113. doi: 10.1038/s41586-021-03403-8, PMID: 33790464 PMC9153763

[ref7] Choi G. B. Dong H.-W. Murphy A. J. Valenzuela D. M. Yancopoulos G. D. Swanson L. W.. (2005). Lhx6 delineates a pathway mediating innate reproductive behaviors from the amygdala to the hypothalamus. Neuron 46, 647–660. doi: 10.1016/j.neuron.2005.04.011, PMID: 15944132

[ref8] Cross S. K. J. Martin Y. H. Salia S. Gamba I. Major C. A. Hassan S.. (2021). Puberty is a critical period for Vomeronasal organ mediation of socio-sexual behavior in mice. Front. Behav. Neurosci. 14:606788. doi: 10.3389/fnbeh.2020.606788 33551763 PMC7862124

[ref10] Døving K. B. Trotier D. (1998). Structure and function of the vomeronasal organ. J. Exp. Biol. 201, 2913–2925. doi: 10.1242/jeb.201.21.2913 9866877

[ref11] Doyle W. I. Meeks J. P. (2018). Excreted steroids in vertebrate social communication. J. Neurosci. 38:3377. doi: 10.1523/JNEUROSCI.2488-17.2018, PMID: 29519850 PMC5895034

[ref12] Dulac C. Wagner S. (2006). Genetic analysis of brain circuits underlying pheromone signaling. Annu. Rev. Genet. 40, 449–467. doi: 10.1146/annurev.genet.39.073003.093937, PMID: 16953793

[ref13] Falkner A. L. Wei D. Song A. Watsek L. W. Chen I. Chen P.. (2020). Hierarchical representations of aggression in a hypothalamic-midbrain circuit. Neuron 106, 637–648.e6. doi: 10.1016/j.neuron.2020.02.014, PMID: 32164875 PMC7571490

[ref14] Ferrero D. M. Moeller L. M. Osakada T. Horio N. Li Q. Roy D. S.. (2013). A juvenile mouse pheromone inhibits sexual behaviour through the vomeronasal system. Nature 502, 368–371. doi: 10.1038/nature12579, PMID: 24089208 PMC3800207

[ref15] Fraser E. J. Shah N. M. (2014). Complex chemosensory control of female reproductive behaviors. PLoS One 9:e90368. doi: 10.1371/journal.pone.0090368, PMID: 24587340 PMC3938725

[ref16] Haga S. Hattori T. Sato T. Sato K. Matsuda S. Kobayakawa R.. (2010). The male mouse pheromone ESP1 enhances female sexual receptive behaviour through a specific vomeronasal receptor. Nature 466, 118–122. doi: 10.1038/nature09142, PMID: 20596023

[ref17] Haga-Yamanaka S. Ma L. He J. Qiu Q. Lavis L. D. Looger L. L.. (2014). Integrated action of pheromone signals in promoting courtship behavior in male mice. eLife Sci:3. doi: 10.7554/eLife.03025 PMC4107909 25073926

[ref18] Halpern M. Martínez-Marcos A. (2003). Structure and function of the vomeronasal system: an update. Prog. Neurobiol. 70, 245–318. doi: 10.1016/S0301-0082(03)00103-5, PMID: 12951145

[ref19] Hammen G. F. Turaga D. Holy T. E. Meeks J. P. (2014). Functional organization of glomerular maps in the mouse accessory olfactory bulb. Nat. Neurosci. 17, 953–961. doi: 10.1038/nn.3738, PMID: 24880215 PMC4327767

[ref20] Hattori T. Osakada T. Matsumoto A. Matsuo N. Haga-Yamanaka S. Nishida T.. (2016). Self-exposure to the male pheromone ESP1 enhances male aggressiveness in mice. Curr. Biol. 26, 1229–1234. doi: 10.1016/j.cub.2016.03.029, PMID: 27151664

[ref21] He J. Ma L. Kim S. Nakai J. Yu C. R. (2008). Encoding gender and individual information in the mouse Vomeronasal organ. Science 320, 535–538. doi: 10.1126/science.1154476, PMID: 18436787 PMC2602951

[ref22] Holy T. E. (2018). The accessory olfactory system: innately specialized or microcosm of mammalian circuitry? Annu. Rev. Neurosci. 41, 501–525. doi: 10.1146/annurev-neuro-080317-061916, PMID: 29727596

[ref23] Hurst J. L. Beynon R. J. (2004). Scent wars: the chemobiology of competitive signalling in mice. BioEssays 26, 1288–1298. doi: 10.1002/bies.20147, PMID: 15551272

[ref24] Inoue S. Yang R. Tantry A. Davis C.-H. Yang T. Knoedler J. R.. (2019). Periodic remodeling in a neural circuit governs timing of female sexual behavior. Cell 179, 1393–1408.e16. doi: 10.1016/j.cell.2019.10.025, PMID: 31735496 PMC7096331

[ref25] Ishii K. K. Osakada T. Mori H. Miyasaka N. Yoshihara Y. Miyamichi K.. (2017). A labeled-line neural circuit for pheromone-mediated sexual behaviors in mice. Neuron 95, 123–137.e8. doi: 10.1016/j.neuron.2017.05.038, PMID: 28648498

[ref26] Ishii K. K. Touhara K. (2019). Neural circuits regulating sexual behaviors via the olfactory system in mice. Neurosci. Res. 140, 59–76. doi: 10.1016/j.neures.2018.10.009, PMID: 30389572

[ref27] Isogai Y. Si S. Pont-Lezica L. Tan T. Kapoor V. Murthy V. N.. (2011). Molecular organization of vomeronasal chemoreception. Nature 478, 241–245. doi: 10.1038/nature10437, PMID: 21937988 PMC3192931

[ref28] Isogai Y. Wu Z. Love M. I. Ahn M. H.-Y. Bambah-Mukku D. Hua V.. (2018). Multisensory logic of infant-directed aggression by males. Cell 175, 1827–1841.e17. doi: 10.1016/j.cell.2018.11.032, PMID: 30550786 PMC6558521

[ref29] Itakura T. Murata K. Miyamichi K. Ishii K. K. Yoshihara Y. Touhara K. (2022). A single vomeronasal receptor promotes intermale aggression through dedicated hypothalamic neurons. Neuron 110, 2455–2469.e8. doi: 10.1016/j.neuron.2022.05.002, PMID: 35654036

[ref30] Karigo T. Kennedy A. Yang B. Liu M. Tai D. Wahle I. A.. (2021). Distinct hypothalamic control of same- and opposite-sex mounting behaviour in mice. Nature 589, 258–263. doi: 10.1038/s41586-020-2995-0, PMID: 33268894 PMC7899581

[ref31] Karlson P. Lüscher M. (1959). The proposed biological term ‘pheromone’. Nature 183:1835. doi: 10.1038/1831835b0 13622694

[ref32] Kaur A. W. Ackels T. Kuo T.-H. Cichy A. Dey S. Hays C.. (2014). Murine pheromone proteins constitute a context-dependent combinatorial code governing multiple social behaviors. Cell 157, 676–688. doi: 10.1016/j.cell.2014.02.025, PMID: 24766811 PMC4051225

[ref33] Kimchi T. Xu J. Dulac C. (2007). A functional circuit underlying male sexual behaviour in the female mouse brain. Nature 448, 1009–1014. doi: 10.1038/nature06089 17676034

[ref34] Kimoto H. Haga S. Sato K. Touhara K. (2005). Sex-specific peptides from exocrine glands stimulate mouse vomeronasal sensory neurons. Nature 437, 898–901. doi: 10.1038/nature04033, PMID: 16208374

[ref35] Kimoto H. Sato K. Nodari F. Haga S. Holy T. E. Touhara K. (2007). Sex- and strain-specific expression and Vomeronasal activity of mouse ESP family peptides. Curr. Biol. 17, 1879–1884. doi: 10.1016/j.cub.2007.09.042, PMID: 17935991

[ref36] Knoedler J. R. Inoue S. Bayless D. W. Yang T. Tantry A. Davis C.. (2022). A functional cellular framework for sex and estrous cycle-dependent gene expression and behavior. Cell 185, 654–671.e22. doi: 10.1016/j.cell.2021.12.031, PMID: 35065713 PMC8956134

[ref37] Lee H. Kim D.-W. Remedios R. Anthony T. E. Chang A. Madisen L.. (2014). Scalable control of mounting and attack by Esr1+ neurons in the ventromedial hypothalamus. Nature 509, 627–632. doi: 10.1038/nature13169, PMID: 24739975 PMC4098836

[ref38] Lee D. Kume M. Holy T. E. (2019). Sensory coding mechanisms revealed by optical tagging of physiologically defined neuronal types. Science 366, 1384–1389. doi: 10.1126/science.aax8055, PMID: 31831669 PMC7591936

[ref39] Leinders-Zufall T. Lane A. P. Puche A. C. Ma W. Novotny M. V. Shipley M. T.. (2000). Ultrasensitive pheromone detection by mammalian vomeronasal neurons. Nature 405, 792–796. doi: 10.1038/35015572, PMID: 10866200

[ref40] Leypold B. G. Yu C. R. Leinders-Zufall T. Kim M. M. Zufall F. Axel R. (2002). Altered sexual and social behaviors in trp2 mutant mice. Proc. Natl. Acad. Sci. 99, 6376–6381. doi: 10.1073/pnas.082127599, PMID: 11972034 PMC122956

[ref41] Li Y. Dulac C. (2018). Neural coding of sex-specific social information in the mouse brain. Curr. Opin. Neurobiol. 53, 120–130. doi: 10.1016/j.conb.2018.07.005, PMID: 30059820

[ref42] Li Y. Mathis A. Grewe B. F. Osterhout J. A. Ahanonu B. Schnitzer M. J.. (2017). Neuronal representation of social information in the medial amygdala of awake behaving mice. Cell 171, 1176–1190.e17. doi: 10.1016/j.cell.2017.10.015, PMID: 29107332 PMC5731476

[ref43] Lin D. Boyle M. P. Dollar P. Lee H. Lein E. S. Perona P.. (2011). Functional identification of an aggression locus in the mouse hypothalamus. Nature 470, 221–226. doi: 10.1038/nature09736, PMID: 21307935 PMC3075820

[ref44] Liu M. Kim D.-W. Zeng H. Anderson D. J. (2022). Make war not love: the neural substrate underlying a state-dependent switch in female social behavior. Neuron 110, 841–856.e6. doi: 10.1016/j.neuron.2021.12.002, PMID: 34982958 PMC8897222

[ref45] Miller C. H. Campbell P. Sheehan M. J. (2020). Distinct evolutionary trajectories of V1R clades across mouse species. BMC Evol. Biol. 20:99. doi: 10.1186/s12862-020-01662-z, PMID: 32770934 PMC7414754

[ref46] Mombaerts P. (2004). Genes and ligands for odorant, vomeronasal and taste receptors. Nat. Rev. Neurosci. 5, 263–278. doi: 10.1038/nrn1365, PMID: 15034552

[ref47] Mombaerts P. Wang F. Dulac C. Chao S. K. Nemes A. Mendelsohn M.. (1996). Visualizing an olfactory sensory map. Cell 87, 675–686. doi: 10.1016/S0092-8674(00)81387-2 8929536

[ref48] Mueller K. L. Hoon M. A. Erlenbach I. Chandrashekar J. Zuker C. S. Ryba N. J. P. (2005). The receptors and coding logic for bitter taste. Nature 434, 225–229. doi: 10.1038/nature03352, PMID: 15759003

[ref49] Murata K. Touhara K. (2021). Pheromones in Vertebrates. Encycl. Life Sci., 2, 1–10. doi: 10.1002/9780470015902.a0029360

[ref50] Niimura Y. Tsunoda M. Kato S. Murata K. Yanagawa T. Suzuki S.. (2020). Origin and evolution of the gene family of proteinaceous pheromones, the exocrine gland-secreting peptides, in rodents. Mol. Biol. Evol. 25:3389. doi: 10.1093/molbev/msaa220/5907918 PMC7826187 32961551

[ref51] Nikaido M. (2019). Evolution of *V1R* pheromone receptor genes in vertebrates: diversity and commonality. Genes Genet. Syst. 94, 141–149. doi: 10.1266/ggs.19-00009, PMID: 31474650

[ref52] Nodari F. Hsu F.-F. Fu X. Holekamp T. F. Kao L.-F. Turk J.. (2008). Sulfated steroids as natural ligands of mouse pheromone-sensing neurons. J. Neurosci. 28, 6407–6418. doi: 10.1523/JNEUROSCI.1425-08.2008, PMID: 18562612 PMC2726112

[ref53] Osakada T. Abe T. Itakura T. Mori H. Ishii K. K. Eguchi R.. (2022). Hemoglobin in the blood acts as a chemosensory signal via the mouse vomeronasal system. Nat. Commun. 13:556. doi: 10.1038/s41467-022-28118-w, PMID: 35115521 PMC8814178

[ref54] Osakada T. Ishii K. K. Mori H. Eguchi R. Ferrero D. M. Yoshihara Y.. (2018). Sexual rejection via a vomeronasal receptor-triggered limbic circuit. Nat. Commun. 9:4463. doi: 10.1038/s41467-018-07003-5, PMID: 30367054 PMC6203846

[ref55] Pérez-Gómez A. Stein B. Leinders-Zufall T. Chamero P. (2014). Signaling mechanisms and behavioral function of the mouse basal vomeronasal neuroepithelium. Front. Neuroanat. 8:135. doi: 10.3389/fnana.2014.00135, PMID: 25505388 PMC4244706

[ref56] Pfau D. R. Baribeau S. Brown F. Khetarpal N. Marc Breedlove S. Jordan C. L. (2023). Loss of TRPC2 function in mice alters sex differences in brain regions regulating social behaviors. J. Comp. Neurol. 531, 1550–1561. doi: 10.1002/cne.25528, PMID: 37496437 PMC10642801

[ref57] Remedios R. Kennedy A. Zelikowsky M. Grewe B. F. Schnitzer M. J. Anderson D. J. (2017). Social behaviour shapes hypothalamic neural ensemble representations of conspecific sex. Nature 550, 388–392. doi: 10.1038/nature23885, PMID: 29052632 PMC5674977

[ref58] Roberts S. A. Davidson A. J. McLean L. Beynon R. J. Hurst J. L. (2012). Pheromonal induction of spatial learning in mice. Science 338, 1462–1465. doi: 10.1126/science.1225638, PMID: 23239735

[ref9] Silva L. Antunes A. (2017). Vomeronasal Receptors in Vertebrates and the Evolution of Pheromone Detection. Annu. Rev. Anim. Biosci. 5, 353–370. doi: 10.1146/annurev-animal-022516-022801, PMID: 27912243

[ref59] Sheehan M. J. Campbell P. Miller C. H. (2019). Evolutionary patterns of major urinary protein scent signals in house mice and relatives. Mol. Ecol. 28, 3587–3601. doi: 10.1111/mec.15155, PMID: 31232499

[ref60] Stowers L. Holy T. E. Meister M. Dulac C. Koentges G. (2002). Loss of sex discrimination and male-male aggression in mice deficient for TRP2. Science 295, 1493–1500. doi: 10.1126/science.1069259 11823606

[ref61] Tirindelli R. (2021). Coding of pheromones by vomeronasal receptors. Cell Tissue Res. 383, 367–386. doi: 10.1007/s00441-020-03376-6, PMID: 33433690

[ref62] Trouillet A.-C. Keller M. Weiss J. Leinders-Zufall T. Birnbaumer L. Zufall F.. (2019). Central role of G protein Gαi2 and Gαi2+ vomeronasal neurons in balancing territorial and infant-directed aggression of male mice. Proc. Natl. Acad. Sci. USA 116, 5135–5143. doi: 10.1073/pnas.1821492116, PMID: 30804203 PMC6421405

[ref63] Tye K. M. (2018). Neural circuit motifs in valence processing. Neuron 100, 436–452. doi: 10.1016/j.neuron.2018.10.001, PMID: 30359607 PMC6590698

[ref64] van der Lee S. Boot L. M. (1955). Spontaneous pseudopregnancy in mice. Acta Physiol. Pharmacol. Neerl. 4, 442–444. PMID: 13301816

[ref65] Vandenbergh J. G. (1967). Effect of the presence of a male on the sexual maturation of female mice. Endocrinology 81, 345–349. doi: 10.1210/endo-81-2-345 4952008

[ref66] Whitten W. K. (1956). Modification of the oestrous cycle of the mouse by external stimuli associated with the male. J. Endocrinol. 13, 399–404. doi: 10.1677/joe.0.0130399, PMID: 13345955

[ref67] Wong W. M. Cao J. Zhang X. Doyle W. I. Mercado L. L. Gautron L.. (2020). Physiology-forward identification of bile acid–sensitive vomeronasal receptors. Sci. Adv. 6:eaaz6868. doi: 10.1126/sciadv.aaz6868, PMID: 32523992 PMC7259934

[ref68] Wu Z. Autry A. E. Bergan J. F. Watabe-Uchida M. Dulac C. G. (2014). Galanin neurons in the medial preoptic area govern parental behaviour. Nature 509, 325–330. doi: 10.1038/nature13307, PMID: 24828191 PMC4105201

[ref69] Wyatt T. D. (2014). Pheromones and animal behavior: Chemical signals and signatures. 2nd Edn Cambridge: Cambridge University Press.

[ref70] Yang B. Karigo T. Anderson D. J. (2022). Transformations of neural representations in a social behaviour network. Nature 608, 741–749. doi: 10.1038/s41586-022-05057-6, PMID: 35922505 PMC9529293

[ref71] Yang C. F. Shah N. M. (2014). Representing sex in the brain, one module at a time. Neuron 82, 261–278. doi: 10.1016/j.neuron.2014.03.029, PMID: 24742456 PMC4130170

[ref72] Yin L. Hashikawa K. Hashikawa Y. Osakada T. Lischinsky J. E. Diaz V.. (2022). VMHvllCckar cells dynamically control female sexual behaviors over the reproductive cycle. Neuron 110, 3000–3017.e8. doi: 10.1016/j.neuron.2022.06.026, PMID: 35896109 PMC9509472

[ref73] Young J. M. Trask B. J. (2007). V2R gene families degenerated in primates, dog and cow, but expanded in opossum. Trends Genet. 23, 212–215. doi: 10.1016/j.tig.2007.03.004, PMID: 17382427

[ref74] Yu C. R. (2015). TRICK or TRP? What Trpc2−/− mice tell us about vomeronasal organ mediated innate behaviors. Front. Neurosci. 9:221. doi: 10.3389/fnins.2015.00221, PMID: 26157356 PMC4477137

[ref75] Zhao Z. McBride C. S. (2020). Evolution of olfactory circuits in insects. J. Comp. Physiol. A 206, 353–367. doi: 10.1007/s00359-020-01399-6, PMID: 31984441 PMC7192870

